# Irregular-Shaped Hematoma Predicts Postoperative Rehemorrhage After Stereotactic Minimally Invasive Surgery for Intracerebral Hemorrhage

**DOI:** 10.3389/fneur.2022.727702

**Published:** 2022-03-11

**Authors:** Likun Wang, Sheng Luo, Siying Ren, Hui Yu, Guiquan Shen, Guofeng Wu, Qingwu Yang

**Affiliations:** ^1^Affiliated Hospital of Guizhou Medical University, Guiyang, China; ^2^Second Affiliated Hospital of Army Military Medical University, Chongqing, China

**Keywords:** intracerebral hemorrhage, stereotactic minimally invasive surgery, regular-shaped hematoma, irregular-shaped hematoma, postoperative rehaemorrhage

## Abstract

**Background and Purpose:**

Minimally invasive surgery (MIS) is performed to treat patients with intracerebral hemorrhage (ICH) with favorable results. However, postoperative rehemorrhage is a significant risk. The present study retrospectively analyzed the association of irregular-shaped hematoma with postoperative rehemorrhage following stereotactic MIS (sMIS).

**Methods:**

We enrolled 548 patients with spontaneous ICH who underwent sMIS. Based on the hematoma shape, the patients were assigned to the regular-shaped hematoma group (RSH group; 300 patients) or irregular-shaped hematoma group (ISH group; 248 patients). Logistic regression analysis was performed to identify the predictors of postoperative rehemorrhage after sMIS for ICH evacuation. The functional outcome was assessed using the modified ranking scale (mRS) score at discharge. A receiver operating characteristic (ROC) curve was used to confirm the results.

**Results:**

Among 548 patients with ICH who underwent sMIS, 116 developed postoperative rehemorrhage. Postoperative rehemorrhage occurred in 30.65% of patients with ISH and 13.30% with RSH (*P* < 0.01), with a significant difference between the ISH and RSH groups. Among 116 patients with postoperative rehemorrhage, 76 (65.52%) showed ISH on CT scan. In 432 patients without postoperative rehemorrhage, only 39.81% displayed ISH. The logistic regression analysis demonstrated that ISH could independently predict postoperative rehemorrhage. The sensitivity, specificity, positive predictive value, and negative predicative value were 0.655, 0.398, 0.655, and 0.602, respectively. The ROC analysis confirmed the value of ISH in predicting postoperative rehemorrhage with an area under the curve of 0.629.

**Conclusions:**

Irregular-shaped hematoma was an independent predictor of postoperative rehemorrhage after sMIS.

## Introduction

Spontaneous intracerebral hemorrhage (sICH) is associated with high mortality and disability worldwide. There are few established treatment strategies for sICH that improve neurological outcomes (1, 2). Hematoma volume is a major determinant of outcome in patients with ICH (3). Previous studies have demonstrated that the extent of ICH-mediated brain injury is directly related to ICH volume and duration of exposure of brain tissue to blood. Smaller postoperative ICH volumes are associated with improved outcomes and maximum removal of blood predicts a good outcome (4). However, a multicenter randomized surgical trial of ICH did not show an overall advantage of early surgery compared to the initial conservative treatment (5). Early conventional surgery only provided benefits for patients with superficial sICH (6). In recent decades, newer surgical techniques for ICH management, called minimally invasive surgery (MIS), have been evaluated in several large clinical trials (7). The minimally invasive craniopuncture technique is a safe and practical technique that improves the independent survival of patients with small basal ganglia hemorrhage. In addition, stereotactic MIS (sMIS) for ICH evacuation is associated with significantly reduced ICH volume and intraoperative hemorrhage. Catheter-based evacuation of ICH is followed by the administration of recombinant tissue plasminogen activator and causes rapid hematoma lysis and drainage with minimal major adverse events (8). A preliminary study, MISTIE III, showed consistent rates of hematoma evacuation despite technical challenges with the surgical approaches (9). MIS plus alteplase appears to be safe in patients with ICH and may be added to the surgical management of ICH (10). In a recently published study, although MIS did not increase the proportion of patients who achieved a good outcome 365 days after ICH, reduction of the hematoma size to 15 ml or less was associated with improved modified ranking scale (mRS) scores at 365 days in stabilized patients (11). However, postoperative rehemorrhage is a significant challenge in clinical practice. A recent study found that the MIS increased the risk of asymptomatic bleeding (12). Other studies found that the rate of postoperative rehemorrhage or rebleeding was almost 26.19% in patients with ICH who underwent MIS and 40% in patients with ICH during the early stage after open surgery (13). The prevention of postoperative rehemorrhage is a possible target for medical intervention because postoperative rehemorrhage is associated with poor outcome in patients with ICH after surgery (14).

Several imaging markers, such as the spot sign, blend sign, and island sign, predict hematoma expansion (HE) (15). The spot sign is a strong predictor of postoperative rehemorrhage after endoscopic surgery for ICH. The blend and black-hole signs on the initial CT scan are associated with postoperative rehemorrhage in patients with ICH following sMIS, which may affect the outcome of patients (16).

Hematoma expansion or growth strongly predicts a worse outcome and can potentially be prevented if high-risk patients are identified and treated early. Previously published studies have demonstrated that irregular-shaped hematoma (ISH) is a strong predictor of HE (17). ISH was independently associated with poor outcomes after ICH, albeit with limited predictive values of an irregular shape (18).

However, few studies have evaluated the correlation of ISH with postoperative rehemorrhage in patients with ICH following sMIS. In the present study, we evaluated whether ISH may be associated with postoperative rehemorrhage following sMIS for ICH evacuation.

## Methods and Procedures

We conducted a retrospective clinical observation study, which was approved by the ethics committee and institutional review board of the Affiliated Hospital of Guizhou Medical University, China. All patients provided informed consent. The study was performed in compliance with the WMA Declaration of Helsinki-Ethical Principles for Medical Research Involving Human Subjects.

### Study Design

We retrospectively collected data from the medical records of patients with ICH treated between January 1, 2018, and December 20, 2020. The study included patients aged over 18 years who had a history of hypertension or had hypertension observed upon admission. The patients had symptoms and signs suggestive of ICH, with supratentorial spontaneous ICH in the basal ganglia, thalamus, subcortex, or cerebral lobe with an ICH volume over 30 ml on a non-enhanced CT scan. The patients were surgical candidates with no contraindications for surgery, and their authorized representatives provided consent for the surgery.

The exclusion criteria were similar to those used in our previously published studies (19). Patients with ICH due to trauma, arteriovenous malformations, aneurysms, anticoagulation therapy, and antiplatelet therapy were excluded. Patients with brainstem, cerebellum, or supratentorial ICH with volumes less than 30 ml, or those patients whose authorized representative did not provide consent for surgery were also excluded.

The patients were diagnosed with ICH using a baseline CT scan performed within 1 h of admission, and the surgery was performed within 24 h after admission. All eligible patients underwent sMIS and were divided into two groups based on their hematoma shape.

### Imaging Analysis

#### Hematoma Shape Assessment

**Figure 1:**
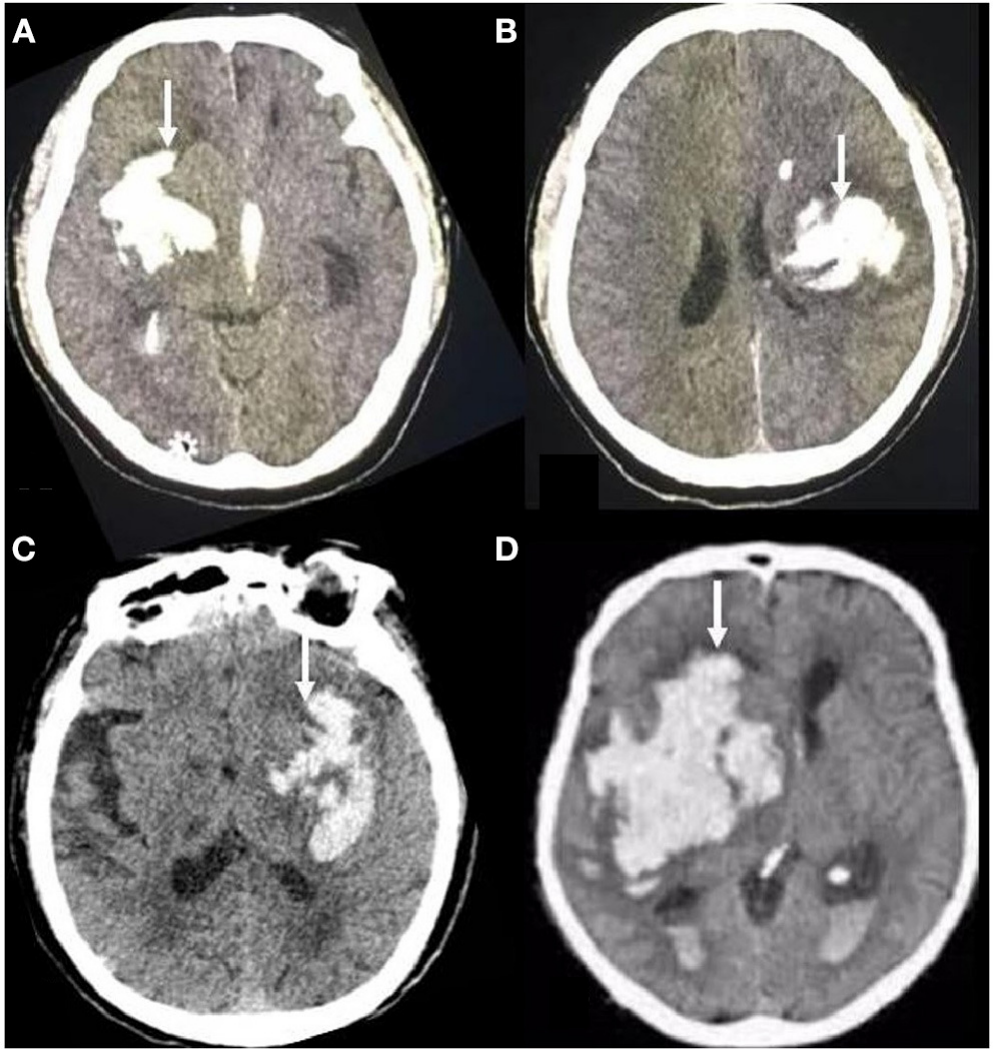
Brain transverse CT section showing examples of hematoma shape. On the transverse CT scans of the brain, the irregular shape of hematoma showed different features, corresponding to grades III **(A, B)** or IV-V **(C, D)** of the Barras scale.

The initial and follow-up CT scans (General Electric Medical Systems, Milwaukee, WI, USA) were performed using standard clinical parameters (axial 3-mm-thick sections, current of 225 mA, window level of 39, and window width of 120). The images were stored for further evaluation. Irregularity of the hematoma shape was rated using the scale established by Barras (20). The scale ranges from grade I (most regular shape) to grade V (most irregular shape). The total rating < grade III indicates shape-regular hematoma. Otherwise, if the total rating ≥ grade III, it indicates the hematoma was irregular (Figure 1). Two experienced neuroimaging experts who were blinded to the clinical information of the patients independently evaluated the hematoma shape by visual inspection (21).

#### Discrepancy Settlement

Discrepancies between the readers about the hematoma shape were settled by discussion. The hematoma shape was determined on the basis of a CT scan repeated before surgery. Surgery was performed after the repeat CT scan, which was performed within 24 h (6–24 h) after admission.

#### Hematoma Volume Calculation

If the CT image slice with the largest hematoma area showed a round or approximately round hematoma shape, the hematoma volume was estimated based on the ABC/2 formula (t = π/6 × l × s × slice) (22). If the hematoma shape was irregular, the volume was calculated using the following formula: V = 2/3Sh (23), where S indicates the area of the largest axial ICH slice on the CT and H indicates the height or depth of the ICH.

#### CT Angiography

If the hematoma on routine CT was suspected to be due to aneurysm or arteriovenous malformation, CT angiography was performed.

### Treatment of the Patients

#### ICH Evacuation by SMIS

The methods used to perform the sMIS procedures were similar to those used in our previous studies (24). Briefly, we fixed a stereotactic instrument on the patient's skull and performed a repeat CT scan to locate the ICH before surgery. After the blood pressure was controlled to a suitable level, we punctured the skull using a 3-mm-diameter needle (with a drill integrated into the needle guard) under the guidance of the stereotactic instrument. After the drill was replaced by a tip-blunt plastic-needle core, the LY-1-type puncture-needle set was slightly advanced into the hematoma. Following the removal of the plastic-needle core, the liquid part of the hematoma was aspirated using a 10-ml syringe and a plastic tube connected to the needle guard. Aspiration was stopped when the first resistance was encountered, and the needle guard connected to a plastic tube was retained in place for several days for drainage. After removing the location framework and stereotactic apparatus, the patients were transferred to the intensive care unit. Then, 50,000 units of urokinase (diluted in 2 ml of normal saline) were slowly injected every 8 h into the residual hematoma to dissolve the solid component of the hematoma. The needle system was closed for 2 h before reopening to allow spontaneous drainage. The first postoperative follow-up CT scan was performed on the day after the surgery, and the second postoperative CT was performed on the third day after surgery. Some patients required a third or even a fourth postoperative follow-up CT scan. The LY-1-type puncture-needle system was removed after the ICH was either completely or nearly completely removed. A repeat CT scan was performed at any time after surgery if the patients showed neurological deterioration.

#### Medications

All the patients in our study received standard medical management based on the guidelines for the treatment of hypertensive ICH (25), including management of coagulopathy, blood pressure, secondary brain injury, and intracranial pressure. In addition, patients received supportive treatment, including prevention of deep venous thrombosis, control of temperature and blood glucose, nutritional support, and prevention of other complications.

### Criteria for Postoperative Rehemorrhage

**Figure 2:**
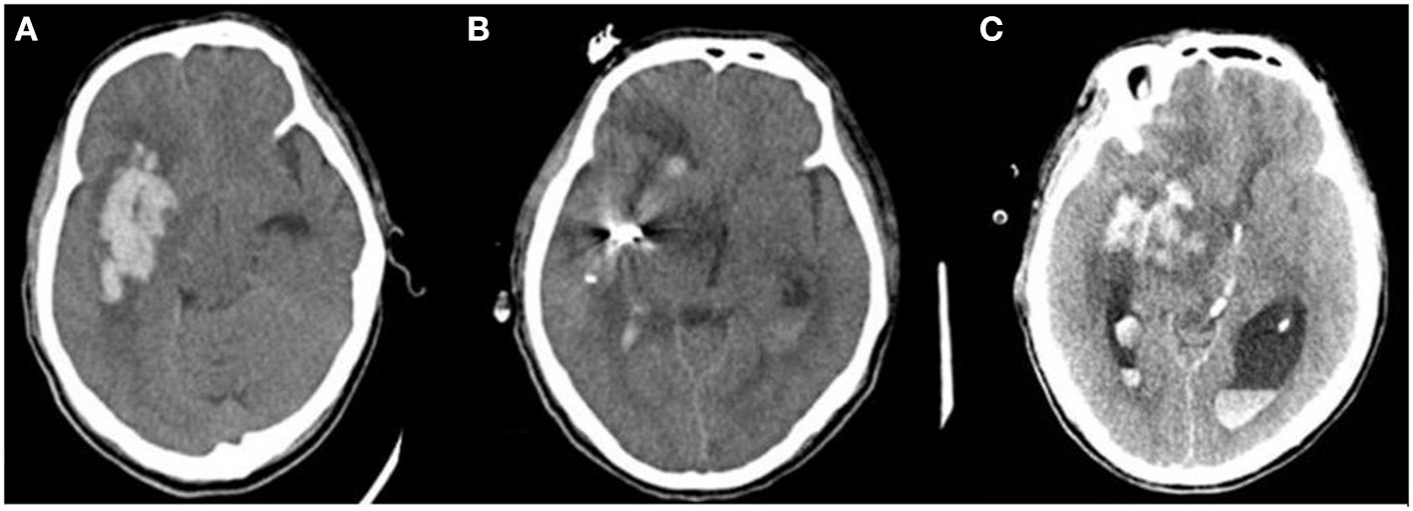
Postoperative rehemorrhage in patients with ISH following sMIS. Postoperative rehemorrhage occurred in patients with irregular-shaped hematoma. **(A)** hematoma before surgery; **(B)** Hematoma size significantly decreased on the first postoperative follow-up CT scan; **(C)** Hematoma size increased on the second follow-up CT scan compared to the previous CT scan, suggesting postoperative rehemorrhage.

Postoperative rehemorrhage was defined as the reappearance of the hematoma or hyperdensity in the hematoma region on any follow-up CT scan after it was completely removed (Figure 2), as confirmed by the previous CT scan (26). An increase in the hematoma volume of >33% or 12.5 ml (27) compared to the ICH volume determined using the previous CT scan minus the ICH volume removed by the operation was also considered postoperative rehemorrhage.

### Statistical Analysis

On the basis of the assumption that 38% of the patients with ICH would have an mRS score of 0–3 following sMIS (28), we estimated that 180 participants would provide 95% statistical power at an α level of 0.05. The permissible error β was 0.1, and the inspection efficiency was 0.9. The sample size was calculated using the following formula:


$$\begin{array}{l} N\;=\;{\frac{z_{\alpha }^{2}p\left(1\;-\;p\right)}{\delta }}^{2} \end{array}$$

A commercially available software package (version 22.0; SPSS Statistical software; IBM Corp., Armonk, NY, USA) was used to perform the statistical analyses. Categorical data are shown as a number (percentage) and continuous data as x̄±S or median and interquartile range (IQR, 25th−75th percentile). Demographic, clinical, and radiological characteristics were compared using Student's *t-*tests (for normal distribution) or a non-parametric test (if the data were not normally distributed). Predictors of postoperative rehemorrhage were evaluated using a univariable logistic regression model, and possible predictors with a *p*-value <0.05 were included in the multivariable analysis for assessment of independent predictors. Receiver operating characteristic curve (ROC) analysis was performed to assess the value of ISH in predicting postoperative rehemorrhage. The interobserver reliability of ISH and regularity was assessed by calculating the κ values. The κ values were categorized as previously reported (29). A κ value of 1 indicated total agreement between the observers. A *p*-value <0.05 was considered to indicate a statistically significant difference.

## Results

### Participants

**Figure 3:**
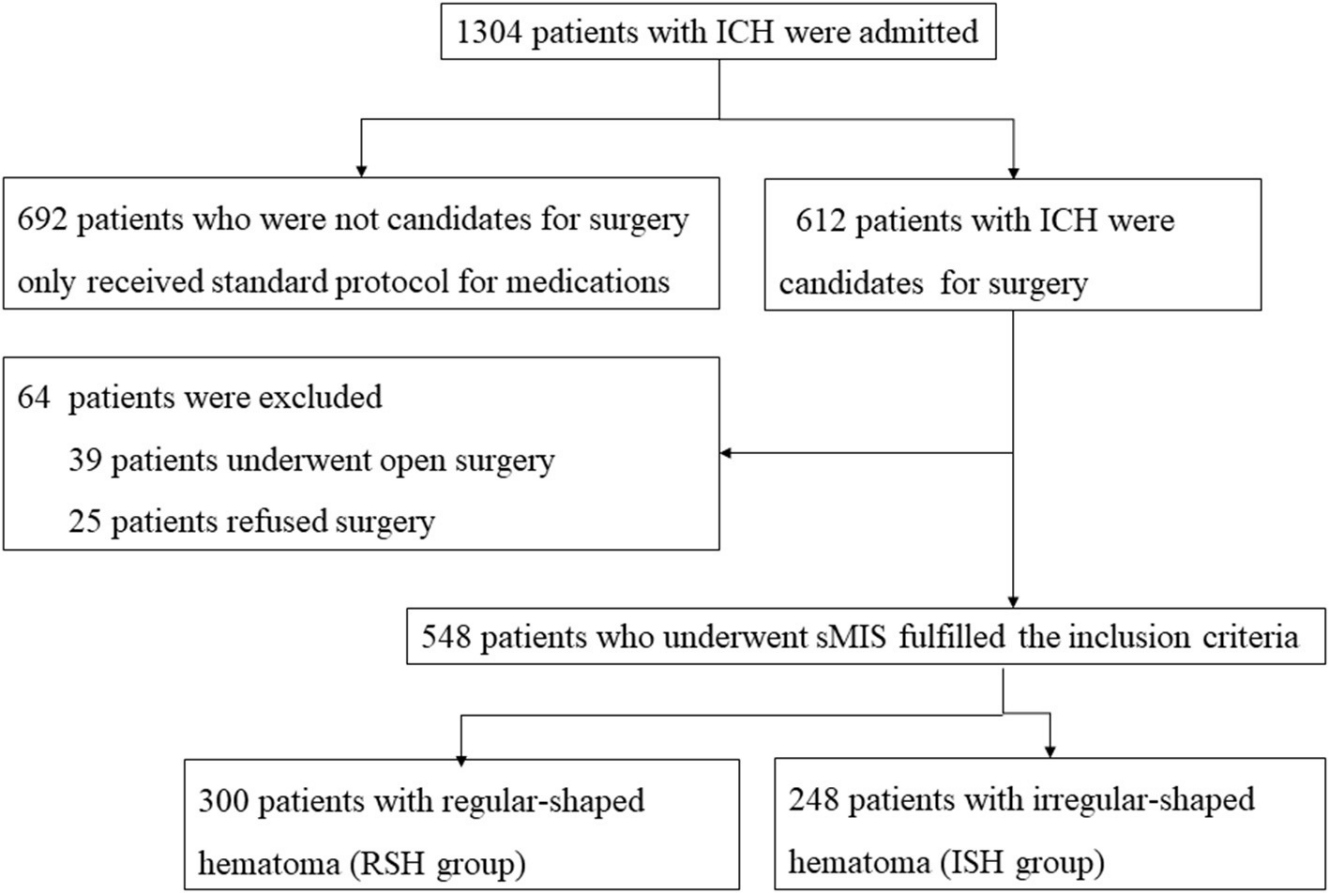
Flowchart of patients‘ inclusion. During the study period, 1,034 patients with ICH were admitted to the Affiliated Hospital of Guizhou Medical University. Among them, 548 patients underwent stereotactic minimally invasive surgery for ICH evacuation. The irregular-shaped hematoma was found in 248 patients and regular-shaped hematoma in 300 patients.

Between January 1, 2018, and December 30, 2020, 1,304 patients with sICH were admitted to the Affiliated Hospital of Guizhou Medical University. Among them, 548 patients underwent sMIS, 39 underwent craniotomy for ICH clearance, 25 refused surgery, and the remaining 692 received standard medical management (Figure 3).

A total of 548 patients fulfilled the inclusion criteria. Complete data were available for all patients. Among the 548 patients, 300 fulfilled the criteria for hematoma shape grade I–II, rated by the scale created by Barras (20), and were classified as the regular–shaped hematoma (RSH) group. The remaining 248 patients with hematoma shape grade ≥ III were assigned to the ISH group (Figure 1).

### Interobserver Agreement in Hematoma Shape

Among the 548 included patients, 248 had an irregularly-shaped hematoma on repeat preoperative CT. The remaining 300 patients had hematoma with a regular shape. Discrepancies were observed in only 13 patients between the two neuroimaging experts, which were settled by discussion (a κ value was 0.962, *P* < 0.001). The interobserver agreement for identifying the hematoma shape was excellent.

### Baseline Data of the Included Patients

Between January 1, 2018, and December 30, 2020, 1,034 patients were assessed for eligibility and 548 fulfilled the inclusion criteria. Among the 548 participants, 392 were men and 156 were women. The ages ranged from 33 to 90 years (average: 58.65 ± 12.36) and 344 patients had a history of hypertension. The time from symptom onset to baseline CT was 5.5 (range: 2–11) h. The admission GCS score was 10.08 ± 3.49 and the NIHSS score was 17.27 ± 5.16.

Based on the hematoma shape, patients were categorized in the ISH (248 patients) or the RSH (300 patients) group. No significant differences were noted between the two groups in terms of age, history of smoking, alcohol use, hypertension, preoperative ICH volume, anticoagulant use, GCS score at admission, NIHSS score at admission, time from symptom onset to baseline CT, or time from symptom onset to surgery (Table 1).

**Table 1:** Comparison of baseline data between regular-shaped and irregular-shaped hematoma groups.

**Factors**	**ISH group (248)**	**RSH group (300)**	**χ^2^/F**	***P-*value**
Ages (years, x±s)	58.35 ± 12.30	58.06 ± 12.87	−0.27	0.79
Gender (male,%)	171 (68.95%)	221 (73.67%)	1.48	0.22
History of smoking (*n*,%)	117 (47.18 %)	146 (48.67%)	0.12	0.72
History of drinking (*n*,%)	103 (41.53%)	131 (43.67%)	0.25	0.62
History of hypotension (*n*,%)	153 (61.69%)	191 (63.67%)	0.23	0.63
History of DM	11 (4.44%)	13 (4.33%)	0.83	0.66
Anticoagulants (*n* = 253,%)	4 (4.11%)	3 (1.92%)	1.08	0.30
Hematoma volume (ml, IQR)	35.81 ± 20.25	36.65 ± 20.43	−0.48	0.63
Systolic pressure (mmHg, x±s)	171.79 ± 30.93	172.38 ± 27.75	−0.24	0.81
Diastolic pressure (mmHg, x±s)	100.17 ± 20.04	100.58 ± 19.33	−0.24	0.55
GCS on admission (points, IQR)	11 (8, 13)	11 (8, 13)	−1.32	0.19
NIHSS on admission (points, IQR)	15 (12.25, 18)	15 (12, 18.75)	27.04	0.71
Time for baseline CT (h, IQR)	5.75 (3, 10)	5 (2, 11)	−0.41	0.68
Time from onset to surgery (h, IQR)	17 (10, 35)	17.5 (10, 31.5)	−1.37	0.17
Duration of surgery (h, IQR)	1.5 (1.0, 2.0)	1.5 (1.0, 2.0)	−0.18	0.86
Time for removing the tube (days, IQR)	4.53 ± 3.01	4.70 ± 2.33	−2.52	0.01
ICH ruptured into the venrticles (*n*, %)	83 (33.47%)	107 (35.67%)	0.29	0.59
Postoperative rehaemorrhage (n,%)	76 (30.65)	40 (13.33)	24.38	0.00
Poor functional outcome (n, %)	173 (69.76)	55 (18.33)	51.50	0.00

*ISH, irregular-shaped hematoma; RSH, regular-shaped hematoma; GCS, glasgow coma scale; NIHSS, national institute of health stroke scale*.

### Changes in ICH Volume After Surgery and Postoperative Rehemorrhage

No difference in the ICH volume was noted between the ISH and RSH groups before the surgery. A significant difference in the residual ICH volume after surgery was observed between the two groups (*p* = 0.001), showing that the hematoma shape influenced ICH removal (Table 2). Among the 548 patients who underwent sMIS, 116 (21.16%) had postoperative rehemorrhage.

**Table 2:** Changes in hematoma volume before and three days after surgery.

**Group**	**Preoperative ICH volume (ml, IQR)**	**Postoperative Residual ICH volume (ml, IQR)**	**Time for removing the tube (days, IQR)**
SIH group (*n =* 248)	33 (20–47)	3 (1–6.25)^&^	5.29 ± 2.86
SRH group (*n =* 300)	33 (22.25–47)	2 (1–4)	4.68 ± 2.46
Z (*P*-value)	−0.42 (0.68)	−3.24 (0.001)	−2.667 (0.01)

& *compared with the RSH group, P <0.05 (RSH group: regular-shaped hematoma group, ISH group: irregular-shaped hematoma group)*.

### ISH and Postoperative Rehemorrhage

**Figure 4:**
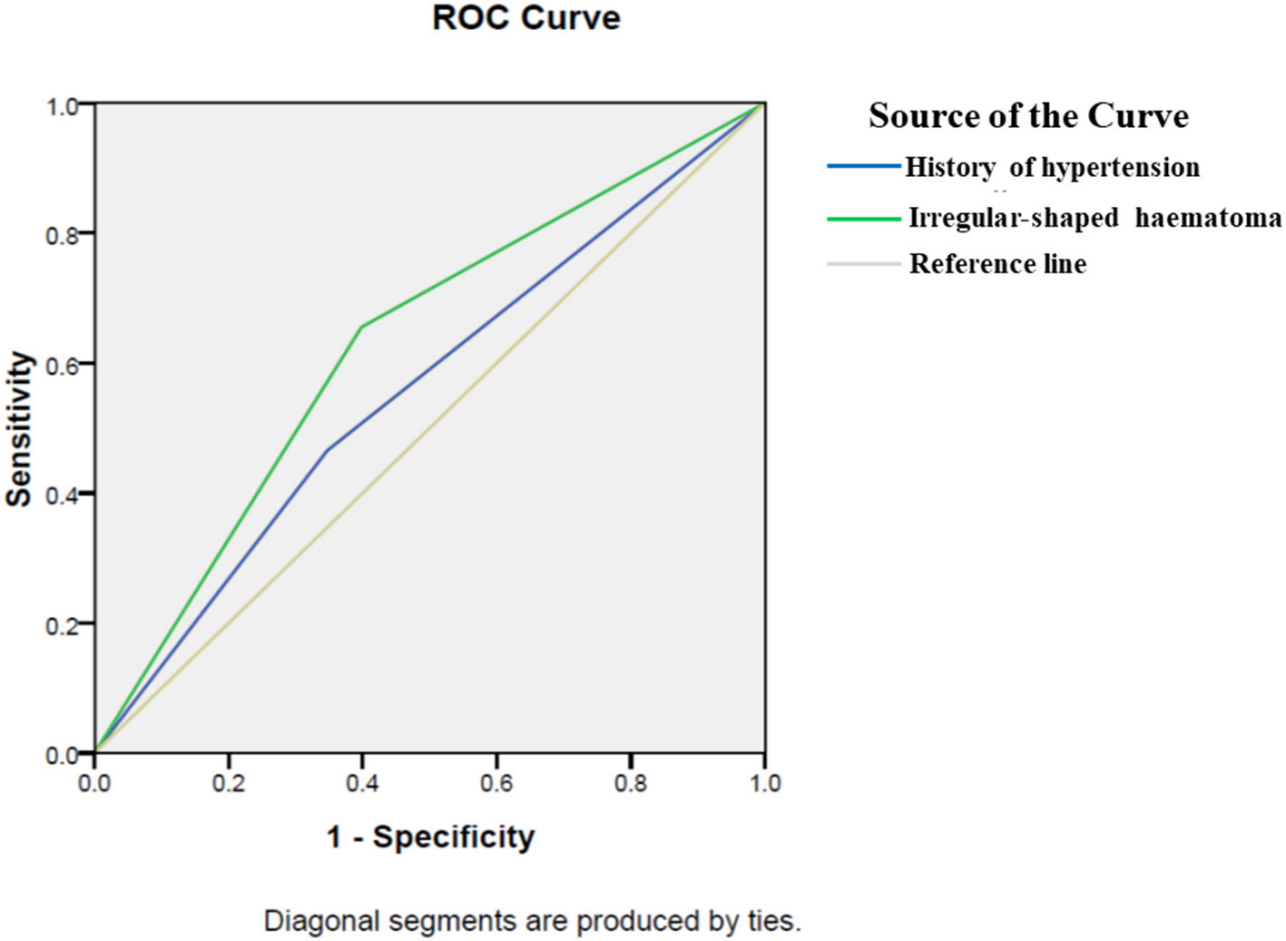
A receiver operating characteristic for predictors of postoperative rehemorrhage. A receiver operating characteristic (ROC) curve was used to confirm the value of irregular-shaped hematoma and history of hypertension in predicting postoperative rehemorrhage following sMIS. The area under the curve of history of hypertension was 0.559(*p* = 0.05). Therefore, a history of hypertension cannot predict postoperative rehemorrhage. However, ROC curve analysis confirmed the value of ISH in predicting postoperative rehemorrhage in patients after sMIS, with an area under the curve of 0.629 (*p* < 0.05).

In 300 patients with RSH, 40 (13.33%) patients developed postoperative hemorrhage. In 248 patients with ISH, 76 (30.65%) patients showed postoperative rehemorrhage (Table 1). The proportion of postoperative hemorrhage was remarkably increased in the ISH group. A significant difference was observed between the ISH and RSH groups (*p* < 0.001). On the other hand, in 116 patients with postoperative rehemorrhage, 76 (65.52%) patients showed ISH on CT scan. However, in 432 patients without postoperative rehemorrhage, only 172 (39.81%) patients displayed ISH (Table 3). These results suggested that patients with ISH were prone to developing postoperative rehemorrhage. To determine the relationship between ISH and postoperative rehemorrhage, we performed a univariate analysis followed by a binary logistic regression. The results showed that ISH was an independent predictor of postoperative rehemorrhage following sMIS (Tables 3, 4). At the same time, a receiver operating characteristic curve (ROC) was used to confirm the value of irregular-shaped hematomain, predicting postoperative rehemorrhage following sMIS with an area under the curve of 0.629 (*p* < 0.05, Figure 4). However, the history of hypertension was protective from postoperative rehaemorrhage (OR = 0.580, *P* = 0.013). The sensitivity, specificity, and positive and negative predictive values of ISH for predicting postoperative rehemorrhage were 0.655, 0.398, 0.655, and 0.602, respectively (Table 5).

**Table 3:** Univariate analysis of predictors related to postoperative rehaemorrhage who underwent stereotactic minimally invasive surgery.

**Factors**	**Postoperative rehaemorrhage positive (*n =* 116)**	**Postoperative rehaemorrhage negative (*n =* 432)**	**χ^2^/F**	***P*-value**
Ages (x±s)	57.46 ± 13.32	58.39 ± 12.41	0.71	0.48
Gender (male, %)	87 (75.00%)	305 (70.60%)	0.87	0.35
History of smoking (*n*,%)	61 (52.59%)	202 (46.76%)	1.24	0.27
History of drinking (*n*,%)	59 (50.86%)	175 (40.51%)	4.01	0.045
History of hypertension (*n*,%)	62 (53.45%)	282 (65.28%)	5.48	0.02
Anticoagulants (*n*,%) *n =* 253	2 (4.23%)	5 (2.41%)	4.32	0.51
History of diabetes (*n*,%)	5 (4.31%)	19 (4.40%)	0.27	0.87
Systolic pressure (mmHg, x±s)	172.64 ± 28.788	171.94 ± 29.346	0.22	0.83
Diastolic pressure (mmHg, x±s)	101.97 ± 23.182	99.97 ± 18.579	0.97	0.33
GCS on admission (points, IQR)	11 (8, 14)	11 (8,13)	18.11	0.80
NIHSS on admission (points, IQR)	15 (12.18.75)	15 (12,18)	35.88	0.45
Time from onset to baseline CT (h, IQR)	3.5 (2.0–9.375)	5.0 (3.0–11.75)	1.56	0.12
ICH volume on admission (ml, IQR)	34.49 ± 21.77	36.75 ± 19.93	1.06	0.29
Hematoma ruptured into ventricles (*n*, %)	40 (34.48%)	150(34.72%)	0.00	0.96
Time from onset to surgery (h, IQR)	23 (10–42)	17 (10–30)	1.61	0.11
Duration of surgery (h, IQR)	1.45 (1.0–1.65)	1.5 (1.0–2.0)	4.45	0.65
Irregular hematoma (n,%)	76 (65.52%)	172 (39.81%)	24.38	0.00
Time for removing the drainage tube	4.87 ± 3.06	4.98 ± 2.56	3.39	0.70
Residual ICH volume	3.0 (2.0–7.5)	2.0 (1.0–5.0)	3.96	0.00
Poor functional outcome	87(75.00%)	141(32.64%)	12.53	0.00

**Table 4:** Binary logistic regression for predictors of postoperative rehemorrhage.

**Variables**	**B**	**Wald**	**OR**	**95%CI**	***P***
Irregular-shaped hematoma	1.08	23.77	2.94	1.91–4.54	0.00
History of drinking	0.52	5.60	1.68	0.93–2.39	0.10
History of hypertension	−0.55	6.17	0.58	0.38–0.89	0.01

**Table 5:** A receiver operating characteristic for predictors of postoperative rehemorrhage.

**Variables**	**Sensitivity predictive values**	**Specificity predictive values**	**Positive predictive values**	**Negative predictive values**	**AUC**	**95%CI**	***P***
Irregular-shaped hematoma	0.655	0.398	0.655	0.602	0.629	0.572–0.685	0.000

### ISH and Poor Outcome

Patients in the ISH group had worse outcomes compared with those in the RSH group (*p* < 0.001). Poor outcomes were seen in 173 patients (69.76%) in the ISH group and 55 patients (18.33%) in the RSH group (Table 1). Among 228 patients with poor outcomes, 166 (72.81%) had ISH and only 62 (27.19%) had RSH.

## Discussion

In the present study, out of 548 patients who underwent sMIS, 116 (21.17%) had postoperative rehemorrhage on follow-up CT scan, which was consistent with previously published studies (30). However, patients with ISH had a significantly higher proportion of postoperative rehemorrhage (30.65%) compared with RSH patients (13.33%). Postoperative rehemorrhage was a severe complication in patients with ICH following surgery, possibly leading to severe secondary damage to the surrounding brain and other medical complications. Patients with postoperative rehemorrhage had a higher rate of poor outcome and increased length of hospital stay. The present study showed that 75.0% of patients with postoperative rehemorrhage had a poor outcome. To confirm the relationship between ISH and postoperative rehemorrhage, we conducted a binary logistic regression analysis, which showed that ISH was an independent predictor of postoperative rehemorrhage after sMIS. Hematoma volume is an important determinant of clinical outcome (3). In patients with ICH who undergo surgery, the outcome is closely associated with postoperative ICH volume (4). Reduction in hematoma size to 15 ml or less was associated with improved mRS scores in stabilized patients. However, hematoma evacuation by open craniotomy has not been found to have beneficial effects in large, randomized trials (13). MIS, including stereotactic catheter aspiration and clearance of ICH with recombinant tissue plasminogen activator, holds promise for the improvement of outcomes of supratentorial brain hemorrhage (29). Sixteen studies, including 1,912 patients, demonstrated that MIS was effective and safe for the treatment of hypertensive ICH, and was associated with decreased mortality and a significantly improved outcome and quality of life in patients compared to other treatment strategies (30). However, postoperative rehemorrhage remains a significant challenge. It is associated with poor outcomes in patients with ICH after surgery (4). Patients with ISH are prone to develop postoperative rehemorrhage, leading to a poor outcome. In the present study, the proportion of patients with poor outcomes was increased in the ISH group compared with the RSH group. Preoperative identification of factors related to postoperative rehemorrhage may be used to guide medical interventions in clinical practice.

Several imaging markers, such as density heterogeneity, blend sign, black hole sign, and margin irregularity on non-contrast CT scans, are associated with hematoma expansion at 24 h. However, the relationships between imaging markers and postoperative rehemorrhage following sMIS remain poorly understood. Previously published studies showed that the CT blend sign and black hole sign are associated with postoperative rehemorrhage (14, 19). However, it is unclear whether ISH is associated with postoperative rehemorrhage. In the present study, we found that patients with ISH are prone to develop postoperative rehemorrhage after sMIS. ISH independently predicts postoperative rehemorrhage in patients who undergo sMIS, and patients with ISH have an increased risk of poor outcomes. The present study also demonstrated that the hypertension history might be protective from postoperative rehemorrhage. Patients with a hypertension history showed a lower percentage of postoperative rehemorrhage (19). In another study, hypertension was not independently associated with postoperative rehemorrhage (31). Combined with these studies, we could postulate that the hypertension history might protect patients with ICH from postoperative rehemorrhage after MIS. However, the present study focused on the effects of irregular-shaped hematoma on the postoperative rehemorrhage after MIS. So, the influence of hypertension history on postoperative rebleeding has not been analyzed in detail.

The outcome of patients with ICH is significantly dependent on the complications (32, 33). Stereotactic aspiration of ICH improves the general condition of patients, but these may induce rehemorrhage (34). In the present study, postoperative rehemorrhage occurred in both RSH and ISH groups. However, the rate of postoperative rehemorrhage in the ISH group was higher than that in the RSH group. Therefore, patients at an increased risk of rehemorrhage should be identified and preoperative measures should be taken to prevent bleeding, such as the application of hemostatic drugs.

In conclusion, ISH increases the risk of postoperative rehemorrhage and leads to a poor outcome in patients with ICH after sMIS. ISH could be used as an independent predictor of postoperative rehemorrhage in patients with ICH who undergo sMIS.

This retrospective study had some limitations. Clinical data were obtained from the medical records; therefore, errors due to the subjective influences of treating physicians could not be completely excluded. Although the ABC/2 formula has been validated to be reliable for ICH volume estimation, it possibly overestimates some irregular shapes of intracerebral hematoma volume. In addition, we were unable to obtain long-term outcomes. These limitations should be addressed in our subsequent study. Certainly, the evaluation of the ICH shape features and rehemorrhage were reliable as they could be identified easily by the naked eye.

## Data Availability Statement

The raw data supporting the conclusions of this article will be made available from the corresponding author by reasonable request.

## Ethics Statement

The studies involving human participants were reviewed and approved by the Ethics Committee of the Affiliated Hospital of Guizhou Medical University approved this retrospective study. The patients/participants provided their written informed consent to participate in this study. Written informed consent was obtained from the individual(s) for the publication of any potentially identifiable images or data included in this article.

## Author Contributions

GW and QY conceived the study, participated in the study design, coordinated the study, and drafted the manuscript. SL, SR, and LW conducted the clinical study. LW performed the statistical analyses and drafted the manuscript. HY and GS are responsible for guiding the analysis of the hematoma shape. All authors read and approved the final manuscript.

## Funding

This research was supported by the Natural Science Foundation of China (81971126), Startup project for High-level overseas talents innovation selection funding (2020)05, and the Guizhou Science and Technology Foundation (No.: Qiankehe support [2021] general [071] and Qiankehe platform talent [2021] general [5612]).

## Conflict of Interest

The authors declare that the research was conducted in the absence of any commercial or financial relationships that could be construed as a potential conflict of interest.

## Publisher's Note

All claims expressed in this article are solely those of the authors and do not necessarily represent those of their affiliated organizations, or those of the publisher, the editors and the reviewers. Any product that may be evaluated in this article, or claim that may be made by its manufacturer, is not guaranteed or endorsed by the publisher.
